# A Parallel Spiking Neural Network Based on Adaptive Lateral Inhibition Mechanism for Objective Recognition

**DOI:** 10.1155/2022/4242235

**Published:** 2022-10-13

**Authors:** Qiang Fu, Hongbin Dong

**Affiliations:** College of Computer Science and Technology, Harbin Engineering University, Harbin 150001, China

## Abstract

Spiking neural network (SNN) has attracted extensive attention in the field of machine learning because of its biological interpretability and low power consumption. However, the accuracy of pattern recognition cannot completely surpass deep neural networks (DNNs). The main reason is that the inherent nondifferentiability of spiking neurons makes SNN unable to be trained directly by the gradient descent algorithm, and there is also no unified training algorithm for SNN. Inspired by the biological vision system, this paper proposes a parallel convolution SNN structure combined with an adaptive lateral inhibition mechanism. And, a way of dynamically evolving the time constant with the training of SNN is proposed to ensure the diversity of neurons. This paper verifies the effectiveness of the proposed methods on static datasets and neuromorphic datasets and extends it to the recognition of breast tumors. Experimental results show that the SNN has obvious advantages in dynamical datasets. For breast tumors, it is also an edge-based task, because the edge of a medical image contains the most important information in the image. This kind of information can provide great help for the noninvasive and accurate diagnosis of diseases. The Experimental results show that the proposed method is very close to the recognition results of DNNs on static datasets, and its performance on neuromorphic datasets exceeds that of DNNs.

## 1. Introduction

In recent years, deep neural network (DNN) has made great progress in the field of computer vision. For instance, super-resolution techniques [1, 2] help to recover image details to medical disease judgment [3], and objective recognition tasks [4] help to segment medical images. However, from the perspective of biological interpretability, a spiking neural network (SNN) is a better choice than a DNN [5, 6], which mimics the activity of biological neurons. Through the simulation of biological neurons, spiking neurons will produce action potential after receiving stimulation. When the action potential reaches the threshold, spiking neurons will fire spikes. Spikes are transmitted along axons and via synapses to postsynaptic neurons, performing the information transmission in SNN.

In realistic scenarios, the correlation between background information and targets should also be considered by SNNs; for example, a person will be classified as a person in the playground, and there is a high possibility of being misidentified in other environments (such as cages). Especially in the field of medical image recognition, background information will greatly affect the judgment of the doctor or model on the lesion region and tumor type. In the field of image processing, the lateral inhibition mechanism [7] is used to avoid the interference of background information on the target region. Lateral inhibition has the following effects: (1) it can enhance contrast and highlight boundaries. When we observe the boundary of a uniformly dark and uniformly bright area, the subjective perception will cause the dark area of the boundary to produce a darker area, and the bright area of the boundary to produce a brighter area. (2) Since the lateral inhibition mechanism can suppress similar information, it can be used as a high-pass filter to suppress the background and low-frequency similar information of the image in space. (3) It has an obvious clustering effect and can fit the subtle discontinuities of the image. Therefore, the lateral inhibition mechanism is an effective way of suppressing background information, and it is consistent with the interpretation of the biological vision system.

The existing SNNs are usually trained by a fixed spiking neuron model. However, in the brain, diversity is one of the most significant features of neurons [8]. Neurons in different brain regions or different types of neurons have different functions and performances. Selecting the membrane-related parameters in SNN according to the empirical value will limit the diversity of neurons. Therefore, different parameter values can be set to affect the membrane potential of the spiking neuron model in SNN, to ensure the diversity of neurons. In addition, most of the existing SNNs adopt the nonparallel architecture, while the architecture of DNNs is relatively complex, which causes the performance of SNNs to be inferior to DNNs on some datasets [9].

In fact, this work includes theory and application. First, we propose a LIF-based evolving spiking neuron model. Secondly, the evolving spiking neuron model is used to design SNN with a parallel structure. Finally, considering the background interference in realistic scenarios, an adaptive lateral inhibition network is proposed. The main contributions of this paper can be summarized as follows:

1. An adaptive lateral inhibition network (LIN) is proposed to suppress interference of background information to detection.
2. Considering that neurons in different brain regions and different types of neurons have different functions and performances, a LIF-based evolving spiking neuron model is proposed. This method provides time constant evolution rules for neurons in different positions in SNN and makes them evolve continuously in the process of network training to ensure the diversity of neurons.
3. A parallel SNN structure is proposed to better extract features from the input information to perform pattern recognition tasks.

The rest of this paper is organized as follows: The related works are provided in Section 2.  Section 3 presents the main contributions, i.e., the adaptive lateral inhibition mechanism, a LIF-based evolving spiking neuron model, and the parallel architecture of SNN. The experimental results are provided in Section 4, which demonstrates the performance of the proposed methods under five different recognition tasks.  Section 5 concludes this paper.

## 2. Related Works

SNNs are generally considered to have advantages in temporal information processing and neuromorphic data processing, and the accuracy of SNNs in the field of edge-task static data object recognition is gradually approaching or even surpassing DNN [10]. Because the edge of a medical image contains the most important information in the image. This kind of information can provide great help for the noninvasive and accurate diagnosis of diseases. Therefore, the SNN can be extended to medical image recognition.

Objective recognition refers to the process in which a special objective (or a type of objective) is distinguished from other objectives (or other types of objectives). In the medical field, the types of diseases can be identified according to medical images. For example, COVID-19 recognition can be performed by computer-aided methods using CT images [11], brain MRI images can be used to recognize brain tumors [12, 13], and the types of breast cancer can be identified according to the histopathological images [14]. In later studies, SNNs were widely used in the fields of object recognition. The approach of [15] considers an SNN model, which is inspired by the model of the local cortical population, as a biological neuro-computing resource for digit recognition was presented. The approach of [16] designs a parallelism network architecture and integrates multiscale spatial information for SNNs. The approach of [17] proposes a method for learning image features with locally connected layers in SNNs using the STDP rule. In this approach, subnetworks compete via inhibitory interactions to learn features from different locations of the input space. The approach of [18] proposes efficient spatiotemporally compressive spike features and presents a lightweight SNN framework that includes a feature extraction layer to extract such compressive features. A convolutional SNN and unsupervised spike-wave time-dependent plasticity (STDP) learning rules are used to classify the malignant melanoma and benign melanocytic nevi skin lesions. Efficient temporal coding, event-driven learning rules, and winner take all (WTA) mechanism jointly ensure sparse spike coding and efficient network learning, with an average accuracy of 83.8% [19]. However, the above-given methods do not consider the interference caused by the background information in realistic scenarios for the recognition task. And, the diversity of neurons is not considered.

Whether neuromorphic data or static data, background information cannot be ignored for object recognition, especially medical images in realistic scenes. Therefore, the lateral inhibition mechanism is an effective means to suppress background information and improve recognition accuracy [20–22]. A lateral inhibition-based Fourier single-pixel imaging technique is proposed to improve the reconstruction contrast and quality for low-contrast scenes [23]. In this study, the original Fourier illumination patterns are replaced with the Fourier illumination patterns based on lateral inhibition for target scanning. This method takes the advantage of quality enhancement and image contrast by using lateral inhibition. The approach of [24] proposed a novel CNN model for single MR image SR tasks, which is motivated by the lateral inhibition mechanism in neurobiology. When the model is lightweight in scale, explicitly imposing inhibitory adjustment on features is considered to help alleviate the representational burden of deep models and improve their SR performance. For improving the quality of the image, based on an improved lateral inhibition network is proposed [25]. To realize enhanced contrast and improved edge definition in images, it built a lateral inhibition network in conjunction with biological visual perception and proposed the adaptive lateral inhibition coefficient.

In the current study, a parallel convolution SNN with the adaptive lateral inhibition machine is proposed, and the adaptive lateral inhibition coefficient adheres to an exponential distribution. Considering the diversity of neurons in SNN, an evolutionary LIF neuron model is proposed.

## 3. Methods

The background information of the image will interfere with the SNN in the process of identifying the object. This paper proposes an adaptive lateral inhibition network (LIN) to solve this problem. In view of the different performance of neurons in different brain regions, a way of evolving membrane potential time constant is proposed to ensure the diversity of neurons. And, a parallel spiking convolution neural network is proposed to enhance the recognition performance.

### 3.1. Adaptive Literal Inhibition Network

**Figure 1:**
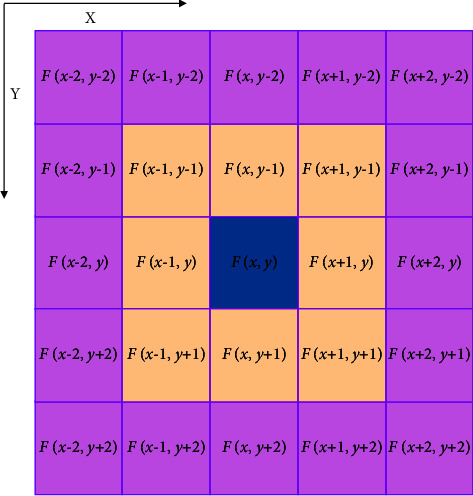
Schematic diagram of lateral inhibition network with radius of 2. F (x, y) is the gray value of the central pixel.

In retinal imaging, the photoreceptor unit in the bright area has a stronger inhibitory effect than that in the dark area. Areas illuminated by light appear brighter and areas illuminated by dark appear darker, thus the contrast can be enhanced. In addition, the intensity gradient in retinal imaging becomes steeper because the closer photoreceptors have stronger inhibition than the farther photoreceptors, which enhances the contrast of the edge. The 2D mathematical model of the traditional LIN can be calculated by the following equation:

(1) $$\begin{array}{c} G\left(u,v\right)=F\left(x,y\right)-\sum_{m=-N}^{N}\sum_{n=-N}^{N}k\left(m,n\right)F\left(x+m,y+n\right)\text{,} \end{array}$$

where *G*(*u*, *v*) is the output information processed by the LIN, F(*x*, *y*) is the pixel of the input image, *k*(*m*, *n*) is the lateral inhibition coefficient of the pixel point(*m*, *n*) to the pixel point (*u*, *v*), *N* is the radius of the inhibition area. The calculation diagram of the side suppression network under radius 2 is shown in Figure 1.

In the traditional LIN model, the selection of lateral inhibition parameter *k*(*m*, *n*) is determined by experience. The lateral inhibition mechanism not only suppresses the background information and enhances the target information but also enhances the noise information in the input image. Therefore, a Butterworth filter is added to the model proposed in this paper to suppress the noise information, and *k*(*m*, *n*) is adjusted exponentially according to the information entropy. It can be calculated by the following equation:

(2) $$\begin{array}{c} G\left(u,v\right)=F_{B}\left(x,y\right)-\sum_{m=-M}^{M}\sum_{n=-N}^{N}k\left(m,n\right)F\left(x+m,y+n\right)\text{,} \end{array}$$

where *F*_*B*_(*x*, *y*) is the output after Butterworth processing, it can be calculated by the following equation:

(3) $$\begin{array}{c} F_{B}\left(x,y\right)=\frac{1}{1+{\left[D\left(x,y\right)/D_{0}\right]}^{2n}}\text{,} \end{array}$$

where *D*_0_ is the cut-off frequency, and $D\left(x,y\right)=\sqrt{x^{2}+y^{2}}$.

The larger the information entropy, the more information the image contains. In the whole image, the focus area accounts for a small proportion, so the amount of information in this area is greater than that in other areas. That is, the amount of background information is less than the amount of target information. The adaptive lateral inhibition coefficient can be calculated by the following equation:

(4) $$\begin{array}{c} k\left(m,n\right)=A\;\exp\;\left(-d_{mn,xy}\cdot H\right)\text{,} \end{array}$$

where *A* is a constant, *d*_*mn*,*xy*_ is the distance between the (*m*, *n*) receptor and the central (*x*, *y*) receptor in one inhibition field. *H* is the information entropy of the image. It can be seen from formula (4) that the smaller the amount of information *H*, the greater the lateral inhibition coefficient *k*, that is, the degree of suppression of the network to the background information is greater than that to the target so that the target area can be highlighted. The information entropy of an input image can be calculated by the following equation:

(5) $$\begin{array}{c} H=\overset{255}{\underset{i=0}{\sum }}P_{xy}\log P_{xy}. \end{array}$$

This method includes not only the aggregation feature of the gray level but also the spatial feature of gray distribution. The adjacent gray value is selected as the spatial feature of the gray distribution. The adjacent gray value and the pixel of the image form a feature tuple, i.e., (*x*, *y*), where *x* is the pixel gray value (0 ≤ *x* ≤ 255), and *y* is the adjacent gray value (0 ≤ *y* ≤ 255). It can be calculated by the following equation:

(6) $$\begin{array}{c} P_{ij}=\frac{f\left(i,j\right)}{N^{2}}\text{.} \end{array}$$

According to formula (4), the larger lateral inhibition coefficient corresponds to the smaller information entropy, so the value of background information entropy is less than that of target information entropy. That is, the inhibition intensity of the adaptive LIN to the background is greater than that to the target.

### 3.2. Spiking Neural Network

The spiking neuron is the basic unit of SNN. In this part, we choose the Leak-Integrate-and-Fire (LIF) neuron [26] with a low computational load as the basic computational unit of SNN. Considering the different performance of neurons in different brain regions, we propose an evolving LIF model with the classification error to form a parallel convolutional spiking neural network.

#### 3.2.1. Evolving Spiking Neuron Model

The back propagation algorithm needs to calculate partial differential. Because the activation function of DNN is continuous and derivative, DNN can use the back propagation algorithm to train the model. Unlike the mature and effective training algorithms such as error backpropagation for DNNs, one of the most difficulties in SNN study is the hardness of training caused by the complex dynamics and nondifferentiable spike activities. The case still remains challenging even if we use the simple LIF neuron model. The basic circuit of the LIF model consists of a capacitor connected in parallel with a resistor. The driving current can be divided into two parts, and the expression formula can be calculated by the following equation:

(7) $$\begin{array}{c} I\left(t\right)=C_{m}\frac{dV_{m}}{dt}+\frac{V_{m}}{R_{m}}\text{,} \end{array}$$

where *C*_*m*_ is the membrane capacitance, *V*_*m*_ is the membrane potential, *R*_*m*_ is the membrane resistance, and *I*(*t*) is the total membrane current. *τ*_*m*_=*R*_*m*_*C*_*m*_ is the time constant of the leakage current. It can be calculated by the following equation:

(8) $$\begin{array}{c} \tau_{m}\frac{dV_{m}}{dt}=-V_{m}\left(t\right)+R_{m}I\left(t\right)\text{.} \end{array}$$

When the neuron receives a constant current stimulation and the membrane is at the resting potential of 0 *mv*, that is, when *I*(*t*)=*I*_0_, the membrane potential can be calculated by the following formula:

(9) $$\begin{array}{c} V_{m}\left(t\right)=R_{m}I_{0}\left[1-\exp\;\left(-\frac{t-t^{\left(0\right)}}{\tau }\right)\right]\text{,} \end{array}$$

where *t*^(0)^ is the time of the last spike. If the value of *V*_*m*_ is less than the firing threshold *V*_*th*_, no spike is generated. On the contrary, if the value of *V*_*m*_ reaches the threshold *V*_*th*_, an output spike is generated at time *t*^(1)^. After the neuron generates a spike, the membrane potential will reach a very low value. Because of this low potential and open ion channel, neurons cannot fire again in a period of time after the previous spike activity. Therefore, the threshold for spike firing can be calculated by the following equation:

(10) $$\begin{array}{c} V_{th}\left(t\right)=R_{m}I_{0}\left[1-\exp\;\left(-\frac{t^{\left(1\right)}-t^{\left(0\right)}}{\tau }\right)\right]\text{.} \end{array}$$

The internal spike time interval can be calculated from the above formulas, that is, the calculation formula of Δ*T*=*t*^(1)^ − *t*^(0)^ is

(11) $$\begin{array}{c} T=\tau_{m}\ln\frac{RI_{0}}{RI_{0}-V_{th}\left(t\right)}. \end{array}$$

Then, the internal spike firing rate can be calculated by the following equation:

(12) $$\begin{array}{c} f=\frac{1}{\left(\tau_{m}\ln RI_{0}/RI_{0}-V_{th}\left(t\right)\right)}\text{.} \end{array}$$

**Figure 2:**
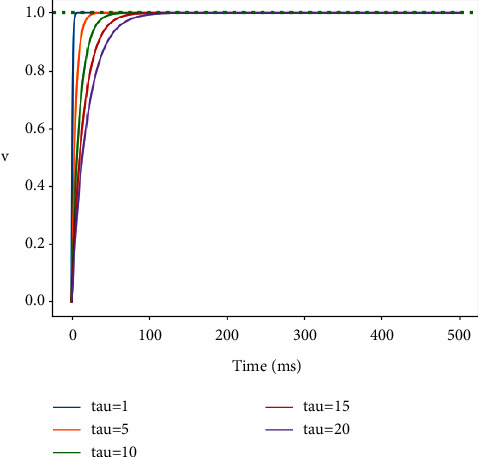
Expression of neuronal membrane potential under different values of time constants.

It can be seen from (12) that the internal spike firing rate of neurons is related to *τ*_*m*_, that is, the internal spike firing rate decreases with the increase of *τ*_*m*_. As shown in Figure 2, the spike firing rate can be adjusted by *τ*_*m*_. In the original LIF model, different neurons also fire at the same rate when the same stimulus is input. This is not the case, the behavior of neurons in different brain regions is different, and the parameters are the same for all neurons, which is not consistent with biological interpretation. Therefore, this paper presents a parameter evolution rule for neurons in different positions in SNN, so that neurons evolve continuously during the network training process. It can be calculated by the following equation:

(13) $$\begin{array}{c} \tau_{m,ji}^{k}=\tau_{m,ji}^{k-1}e^{aE}\text{.} \end{array}$$

*τ*_*m*,*ji*_^*k*−1^ is the parameter value of each neuron in the last training process. Once the measurement error *E* tends to 0, the value of this parameter will tend to the initial value. Parameter *b* is used for control *τ*_*m*_ degree of change. As the measurement error *E* decreases, the value of *τ*_*m*_ will also gradually decrease, so while ensuring the diversity of neurons, it can avoid the oscillation of spike rate in the later stage of training.

#### 3.2.2. Architecture of the SNN

The training of the network relies on the STDP-based backpropagation algorithm. The method is a temporal local learning rule that applies backpropagation to update weight changes at each time step. This approach combines the advantages of accurate gradient descent with the advantages of temporally local efficient STDP. The output layer error function of SNN can be calculated by the following equation:

(14) $$\begin{array}{c} E=\frac{1}{N}\overset{N}{\underset{j=1}{\sum }}\overset{M}{\underset{i=1}{\sum }}{\left(d_{ji}-o_{ji}\right)}^{2}\text{,} \end{array}$$

where *N* is the number of input samples, *M* is the number of neurons in the previous layer. *d*_*ji*_ and *o*_*ji*_ are the desired and actual outputs of neurons, respectively. The change of synaptic weight can be calculated by the following equation:

(15) $$\begin{array}{c} ∆w_{ih}\left(t\right)=\eta \alpha_{i}\left(t\right)\overset{t}{\underset{t^{f}=t-\varepsilon }{\sum }}s_{h}\left(t^{f}\right)\text{,}\\ \alpha_{i}\left(t\right)=\left\{\begin{array}{c} 1,if\;the\;value\;of\;de\;sire\;d\;output\;spike\;is\;1\text{,}\\ -1,if\;the\;value\;of\;de\;sire\;d\;output\;spike\;is\;0\text{,}\\ 0,otherwise\text{,} \end{array}\right. \end{array}$$

where *η* is learning rate and *s*_*h*_(*t*^*f*^) is the spike of a presynapse neuron. Finally, the synaptic weights can be updated by the following equation:

(16) $$\begin{array}{c} w_{ih}\left(t\right)=w_{ih}\left(t\right)+∆w_{ih}\left(t\right)\text{.} \end{array}$$

**Figure 3:**
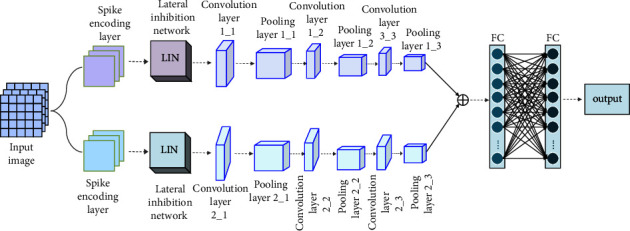
Structure diagram of parallel convolution spiking neural network.

To extract features more effectively, this paper proposes a parallel convolutional SNN to perform pattern recognition tasks. The architecture is mainly composed of a spike encoding layer, an adaptive LIN layer, convolutional layers, pooling layers, and fully connected layers. The spike encoding layer encodes the input information into a spike sequence. The LIN layer is used to suppress the background information and noise of the input pattern. The convolutional layer is the core of the convolutional neural network and is used for feature extraction from lower-level feature maps. The function of the fully connected layer is to connect all the features and send the output value to the classifier for output, as shown in Figure 3.

## 4. Experimental Results

To effectively verify the performance of the network, the proposed method is tested on the static dataset (Fashion-MNIST) [27] and neuromorphic dataset (N-MNIST) [28]. The method is also extended to recognize breast tumors on three different modalities of breast image datasets, namely, breast ultrasound images [29], breast X-ray images [30], and breast histopathological images [31].

The Gaussian distribution hypothesis of classification accuracy is used to calculate the confidence intervals. It can be calculated by the following equation:

(17) $$\begin{array}{c} l=\frac{2*n*p+z-z*\left(\sqrt{z+4*n*p*\left(1-p\right)}\right)}{2\left(n+z\right)}\text{,} \end{array}$$

(18) $$\begin{array}{c} u=\frac{2*n*p+z+z*\left(\sqrt{z+4*n*p*\left(1-p\right)}\right)}{2\left(n+z\right)}\text{,} \end{array}$$

where *l* and *u* are the upper and lower bounds of the confidence interval, respectively, *p* is the classification accuracy, *n* is the sample size, and *z* is the critical value of Gaussian distribution.

### 4.1. Fashion-MNIST Dataset

**Figure 4:**
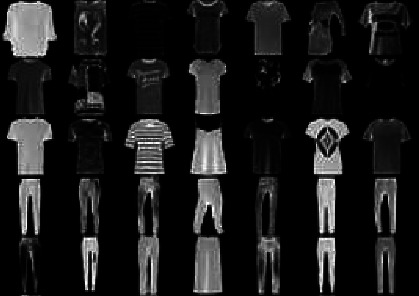
Some sample images of the Fashion-MNIST database [27].

A static image dataset (i.e., Fashion-MNIST [27]) is first used to our method. The Fashion-MNIST dataset is a more challenging classification task than the MNIST dataset. The size of the Fashion-MNIST dataset is the same as the MNIST. It is divided into a training and a test set. The training set receives a randomly selected 6, 000 samples from each class. The remaining samples are used as the training set.  Figure 4 shows some samples of the Fashion-MNIST dataset. The performance comparison of different methods proposed in this paper is tested on the fashion MNIST data set, as shown in Table 1.

As shown in Table 1, it reports the accuracy and confidence intervals of different methods. The nonparallel SNN without LIN achieves 84.03% overall accuracy and the confidence intervals of (0.9456, 0.9541). The nonparallel SNN without LIN achieves 84.26% overall accuracy and the confidence intervals of (0.8353, 0.8496). The nonparallel SNN without LIN achieves 84.72% overall accuracy and the confidence intervals of (0.84, 0.8541). The nonparallel SNN without LIN achieves 94.25% overall accuracy and the confidence intervals of (0.9378, 0.9469). The nonparallel SNN without LIN achieves 94.37% overall accuracy and the confidence intervals of (0.939, 0.948). The 95.0% overall accuracy and the confidence intervals of (0.9456, 0.9541) can be gained by using the parallel SNN with adaptive LIN. The performance of parallel SNN with adaptive LIN is better than that of SNN, SNN with traditional LIN, SNN with adaptive LIN, parallel SNN, and parallel SNN with traditional LIN on the Fashion-MNIST dataset.

Table 2 presents the recognition accuracy of the proposed parallel SNN with adaptive LIN along with some other models on the Fashion-MNIST database. It shows that the results of NALSM8000 [32] report accuracy (98.52%) and confidence interval (0.8514, 0.8651) by using astro-STDP learning rule. The approach of [33] proposed the Voltage-driven Plasticity-centric SNN (VPSNN) which achieved 94.1% classification accuracy on the Fashion-MNIST database. It is a four-step learning model to create a new general learning architecture on the training of SNNs and integrates supervised and unsupervised learning to train an SNN with nondifferential neurons. A supervised SNN model based on the symmetric spike-timing dependent plasticity (sym-STDP) rule is proposed by the approach of [34]. It combines the sym-STDP rule with synaptic scaling and intrinsic plasticity of the dynamic threshold, it achieves 85.31% classification accuracy and (0.846, 0.8599) confidence interval. A binarized spiking neural network (BS4NN) using a direct supervised temporal learning algorithm is proposed by the approach of [35]. BS4NN achieves 87.3% classification accuracy and (0.8663, 0.8794) confidence interval on the Fashion-MNIST dataset. The approach of [36] used the multilayer SNN with the global plasticity and the local learning process, which achieved 89.05% classification accuracy and (0.8842, 0.8965) confidence interval. The approach of [37] used a feedback spiking neural networks, which achieved 90.25% classification accuracy and (0.8965, 0.9082) confidence interval. It proposed a training method that does not rely on the exact reverse of the forward computation. The approaches of [38, 39] both used the spike-based back propagation (BP) rules for classifications. The results of [39] report accuracy (94.38%) by using the Spikingjelly. In this work, the proposed parallel SNN with adaptive LIN outperforms other models by reaching 95.0% recognition accuracy.

### 4.2. N-MNIST Dataset

**Figure 5:**
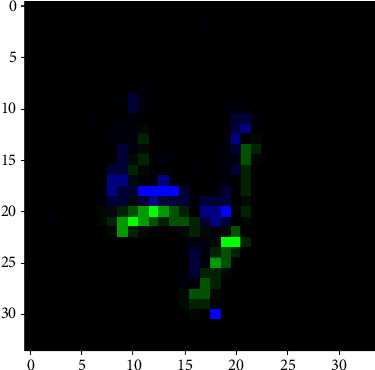
An example of a sample on the N-MNIST neuromorphic dataset.

The proposed method also evaluates on the neuromorphic dataset N-MNIST [28], whose inputs are spikes collected by dynamic vision sensors. It is divided into a training and a test set as the same as the Fashion-MNIST. The training set receives a randomly selected 6, 000 samples from each class. The remaining samples are used as the training set.  Figure 5 shows an example of the N-MNIST dataset. The performance comparison of different methods proposed in this paper is tested on the N-MNIST data set, as shown in Table 3.

Table 3 reports the accuracy and confidence intervals of different methods. The nonparallel SNN without LIN achieves 92.53% overall accuracy and the confidence intervals of (0.92, 0.9303). The nonparallel SNN without LIN achieves 92.61% overall accuracy and the confidence intervals of (0.9208, 0.9311). The nonparallel SNN without LIN achieves 92.61% overall accuracy and the confidence intervals of (0.9208, 0.9311). The nonparallel SNN without LIN achieves 99.52% overall accuracy and the confidence intervals of (0.9936, 0.9964). The nonparallel SNN without LIN achieves 99.56% overall accuracy and the confidence intervals of (0.9941, 0.9967). The 99.67% overall accuracy and the confidence intervals of (0.9954, 0.9976) can be gained by using the parallel SNN with adaptive LIN. The performance of parallel SNN with adaptive LIN is better than that of SNN, SNN with traditional LIN, SNN with adaptive LIN, parallel SNN, and parallel SNN with traditional LIN on the N-MNIST dataset.

Table 4 shows the performance comparison between the proposed method and the state-of-the-art methods on the N-MNIST dataset. It shows that the results of NALSM8000 [32] report accuracy (97.51%) and confidence interval (0.9719, 0.978) by using astro-STDP learning rule. The approaches of [40–43], and [44] used the BPSNN, STBP, DECOLLE, AR-SNN, and SLAYER architectures, respectively. All of them used backpropagation rules for classifications. The approach of [37] used a feedback spiking neural networks, which achieved 99.47% classification accuracy and (0.9931, 0.9959) confidence interval. The approaches of [38, 39] both used the spike-based back propagation (BP) rules for classifications. The results of [39] report accuracy (99.61%) by using the Spikingjelly. In this work, the proposed parallel SNN with adaptive LIN outperforms other models by reaching 99.67% recognition accuracy.

### 4.3. Breast Ultrasound Dataset

**Figure 6:**
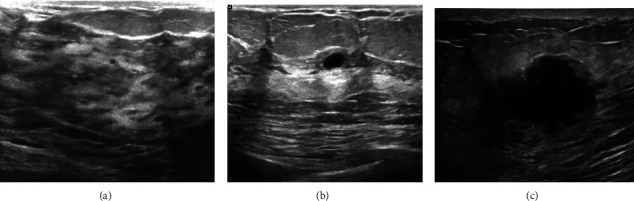
Three types of images in the dataset of breast ultrasound images. (a) Norm. (b) Benign. (c) Malignant.

To further verify the effectiveness of the proposed method, we test it on three different modalities of breast datasets (i.e., ultrasound dataset [29], the Mini-MIAs [30], and the BreaKHis dataset [31]). The breast ultrasound dataset [29], which consists of 780 images. The source images with an average image size of 500 × 500 pixels. It can be categorized into 3 classes, i.e., malignant, benign, and normal tumors, as shown in Figure 6.

**Figure 7:**
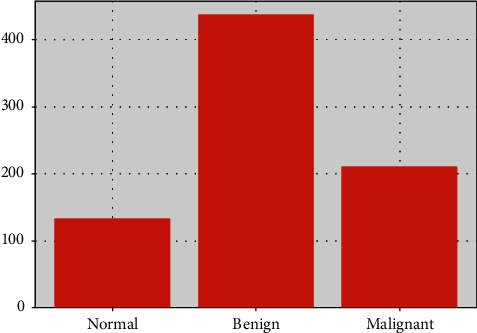
The number of images in each category.

Figure 7 shows the number of images in each category. The dataset contains 133 normal images, 437 benign images, and 210 malignant images. The number of images in a benign category is more than that in the malignant category. To ensure category balance, image enhancement methods are used to expand the number of images. The images of malignant tumors are rotated 90 degrees and 80 degrees respectively, and finally 630 images of malignant tumors are obtained.

**Figure 8:**
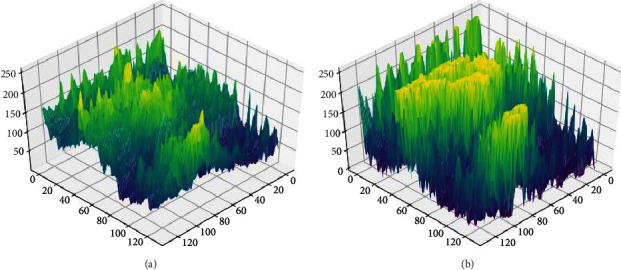
Three-dimensional gray distribution of a benign tumor image. (a) Is processed by the traditional lateral inhibition model. (b) Is processed by the adaptive lateral inhibition model.

Figure 8 shows the three-dimensional gray distribution of a benign tumor image processed by the traditional lateral inhibition model and the adaptive lateral inhibition network respectively. It shows that the target is more distinct to the background after being processed in the proposed algorithm.

As shown in Table 5, it reports the accuracy and confidence intervals of different methods. The 99.2% overall accuracy and the confidence intervals of (0.9758, 0.9972) can be gained by using the parallel SNN with adaptive LIN. The performance of parallel SNN with adaptive LIN is better than that of SNN, SNN with traditional LIN, SNN with adaptive LIN, parallel SNN, and parallel SNN with traditional LIN on the breast ultrasound dataset.

Table 6 shows the performance comparison between the proposed method and the state-of-the-art methods on the breast ultrasound dataset. It shows that accuracies 86.5% can be achieved by using the model of Google AutoML Vision [45]. 85.9% accuracy and 87.8% accuracy can be obtained by using the ResNet-18 (28) [46] and ResNet-18 (224) [46]. Using the ResNet-50 [46], the accuracy of the network is 85.3% and 83.3%. The accuracy of 80.8% and the accuracy of 80.1% are obtained by the Auto-sklearn [47] and AutoKeras [48] respectively. The approach of [49] reports that the accuracy of six different methods (i.e., DAN, DDC, MADA, DAAN_R18, DAAN_R50, and MK_DAAN_R50) is 79.2%, 77.6%, 79.9%, 80.8%, 81.6%, and 83.2%, respectively. Our work can get the best result, i.e., 99.2%, on the BreastMNIST database.

### 4.4. Mini-MIAS Dataset

**Figure 9:**
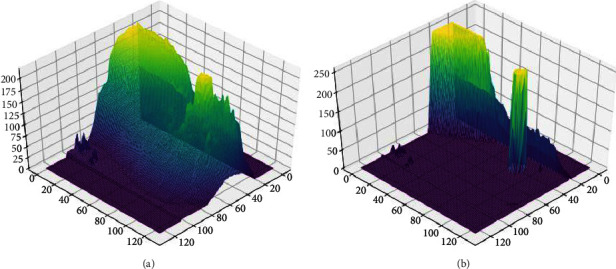
Three-dimensional gray distribution of a malignant tumor image. (a) Is processed by the traditional lateral inhibition model. (b) Is processed by the adaptive lateral inhibition model.

The Mammographic Image Analysis Society (MIAS) dataset includes breast X-ray image of 161 patients. This work uses the mini-MIAs database, which contains images with a size of 1024 × 1024 pixels [30].  Figure 9(a) shows the three-dimensional gray distribution of a malignant tumor image which processed by the traditional lateral inhibition model.  Figure 9(b) is the three-dimensional gray distribution which processed by the adaptive lateral inhibition model. It shows that the target region is more distinct to the background after being processed in the proposed algorithm.

As shown in Table 7, it reports the accuracy and confidence intervals of different methods. The 98.99% overall accuracy and the confidence intervals of (0.9556, 0.9966) can be gained by using the parallel SNN with adaptive LIN. The performance of parallel SNN with adaptive LIN is better than that of SNN, SNN with traditional LIN, SNN with adaptive LIN, parallel SNN, and parallel SNN with traditional LIN on the Mini-MIAS dataset.

Table 8 shows the performance comparison between the proposed method and other methods on the Mini-MIAS dataset. The method of adaptive thresholding provides 93% accuracy in the approach of [50]. An accuracy of 94.57% is achieved in [51] using Fisher linear discriminant analysis features of neighborhood structural similarity. An accuracy of 96.7% is obtained in the approach of [52] using deep distance metric learning with the stochastic gradient descent algorithm. Using texture features with a neural network classifier provides 95.2% accuracy [53]. Super-resolution reconstruction module with texture features provides 96.7% accuracy in [54]. The approach of [55] reports that the accuracy of using different deep learning methods (i.e., GoogLeNet, ResNet-101, DenseNet-201, Xception, Inception-v3, and DisepNet) is 94.67%, 94.65%, 94.47%, 95.17%, 94.51%, and 95.60%, respectively. In our work, the proposed parallel SNN with adaptive LIN outperforms other models by reaching 98.99% recognition accuracy.

### 4.5. BreaKHis Dataset

**Figure 10:**
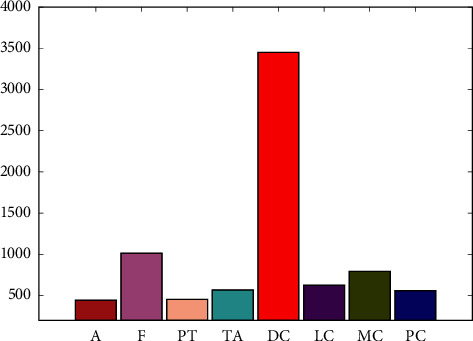
The detailed information of the BreaKHis database.

The BreaKHis dataset [31] contains eight different types of breast cancer images, as shown in Figure 10. Because the number of ductal carcinoma is much more than other types, the other seven kinds of images are rotated and expanded at different angles to maintain category balance.

**Figure 11:**
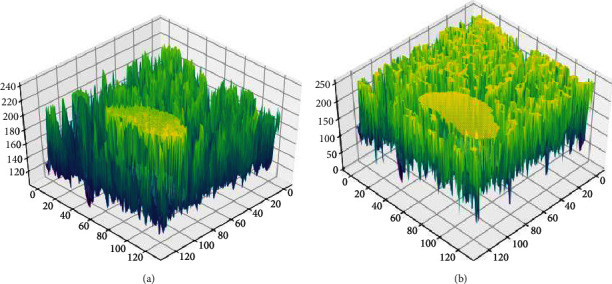
Three-dimensional gray distribution of a malignant tumor (papillary) image. (a) Is processed by the traditional lateral inhibition model. (b) Is processed by the adaptive lateral inhibition model.

Figure 11 shows the three-dimensional gray distribution of a malignant tumor (papillary) image processed by the traditional lateral inhibition model and the adaptive lateral inhibition network respectively. It shows that the target is more distinct to the background after being processed in the proposed algorithm.

As shown in Table 9, the accuracy of different methods is reported. The 97.85% overall accuracy can be gained by using the parallel SNN with adaptive LIN. The performance of parallel SNN with adaptive LIN is better than that of SNN, SNN with traditional LIN, SNN with adaptive LIN, parallel SNN, and parallel SNN with traditional LIN on the BreaKHis dataset.

Table 10 shows the performance comparison between the proposed method and several other studies on the BreaKHis dataset. The results of [56] report accuracies (86%–90%) by using the AlexNet. The experimental results of [31] demonstrated that the QDA classifier can get higher accuracies than RF and SVM classifiers on BreaKHis. A deep convolutional neural network is used to achieve 95.7%–97.1% classification accuracies in [57]. The approach of [58] investigated the performance of five CNNs architectures (i.e., LeNet-5, AlexNet, VGG-16, ResNet-50, and Inception-v1) on the basis of test accuracy. The Inception-v1 can achieve the test accuracy of 89%, 92%, 94%, and 90%, respectively, at 40×, 100×, 200× and 400× magnification factor classification. The approach of [59] proposed an efficient and lightweight CNN model for histopathological image classification based on MobileNet. It achieves the test accuracy of 91.42%, 89.93%, 92.70%, and 85.84%, respectively, at 40×, 100×, 200× and 400× magnification factor classification. The ensemble SNN [10] can achieve 98.7%, 95.1%, 96.7%, and 97.5% accuracies, respectively, which are higher than other approaches. The result of our work achieves 97.06%, 97.29%, 98.60%, and 98.45% accuracies, respectively, at 40×, 100×, 200× and 400× magnification factor classification. The proposed method is not the best on the magnification of 40×, however, it is better than others on the 100×, 200× and 400× images.

## 5. Conclusions and Discussion

A parallel SNN architecture, underpinned by the STDP-based backpropagation learning rule and evolving spiking neurons, has been presented in this paper. To overcome the interference of background information and improve the performance of the SNN, an adaptive lateral inhibition mechanism was proposed. Through verification on different datasets, the accuracy of the proposed method is improved. Because the Fashion-MNIST and the neuromorphic datasets are relatively clear, it is hardly affected by the background information, so the improvement is not obvious. For medical image data sets in the real world, the method proposed in this paper improves the classification accuracy relatively significantly. From the perspective of recognition performance, the proposed SNN outperforms other machine learning models in this paper. Especially on the neuromorphic dataset, SNN has inherent advantages. Because the neuromorphic data contains more dynamic temporal information, and the spike event is naturally compatible with the signal format in the neural network. For static datasets, SNN usually needs to optimize or expand the model to reach the same level as DNN. However, such an approach would make SNN lose its biological interpretability. Future work will focus on the research of SNN training algorithm, so that SNN can be applied to more realistic tasks.

## Figures and Tables

**Table 1:** Performance comparison of SNN, SNN with traditional LIN, SNN with adaptive LIN, parallel SNN, parallel SNN with traditional LIN, and parallel SNN with adaptive LIN on the Fashion-MNIST dataset (*z* = 1.96).

Models	Accuracy (%)	Confidence intervals
Nonparallel SNN without LIN	84.03	(0.833, 0.8473)
Nonparallel SNN with traditional LIN	84.26	(0.8353, 0.8496)
Nonparallel SNN with adaptive LIN	84.72	(0.84, 0.8541)
Parallel SNN without LIN	94.25	(0.9378, 0.9469)
Parallel SNN with traditional LIN	94.37	(0.939, 0.948)
Parallel SNN with adaptive LIN	95.0	(0.9456, 0.9541)

**Table 2:** Performance comparison between the proposed method and the state-of-the-art methods on the Fashion-MNIST dataset (*z* = 1.96).

Models	Algorithms	Accuracy (%)	Confidence intervals
NALSM8000 [32]	Astro-STDP, GD on last layer	85.84	(0.8514, 0.8651)
VPSNN [33]	Equi-prop, STDP	82.69	(0.8194, 0.8342)
Supervised-SNN [34]	sym-STDP	85.31	(0.846, 0.8599)
BS4NN [35]	Temporal backpropagation	87.3	(0.8663, 0.8794)
GLSNN [36]	Global feedback alignment, STDP	89.05	(0.8842, 0.8965)
FSNN [37]	IDE-LIF	90.25	(0.8965, 0.9082)
LISNN [38]	Spike-based BP	92.07	(0.9152, 0.9258)
Spikingjelly [39]	Spike-based BP	94.38	(0.9391, 0.9481)
This work	STDP-based BP	95.0	(0.9456, 0.9541)

**Table 3:** Performance comparison of SNN, SNN with traditional LIN, SNN with adaptive LIN, parallel SNN, parallel SNN with traditional LIN, and parallel SNN with adaptive LIN on the N-MNIST dataset (*z* = 1.96).

Models	Accuracy (%)	Confidence intervals
Nonparallel SNN without LIN	92.53	(0.92, 0.9303)
Nonparallel SNN with traditional LIN	92.61	(0.9208, 0.9311)
Nonparallel SNN with adaptive LIN	92.61	(0.9208, 0.9311)
Parallel SNN without LIN	99.52	(0.9936, 0.9964)
Parallel SNN with traditional LIN	99.56	(0.9941, 0.9967)
Parallel SNN with adaptive LIN	99.67	(0.9954, 0.9976)

**Table 4:** Performance comparison between the proposed method and the state-of-the-art methods on the N-MNIST dataset (*z* = 1.96).

Models	Algorithms	Accuracy (%)	Confidence intervals
NALSM8000 [32]	Astro-STDP, GD on last layer	97.51	(0.9719, 0.978)
BPSNN [40]	Backpropagation	98.74	(0.985, 0.9894)
STBP [41]	Spatial and temporal backpropagation	98.78	(0.9855, 0.9898)
DECOLLE [42]	Backpropagation	96.0	(0.956, 0.9637)
AER-SNN [43]	Backpropagation	96.3	(0.9591, 0.9665)
SLAYER [44]	Backpropagation	98.89	(0.9867, 0.9908)
FSNN [37]	IDE-LIF	99.47	(0.9931, 0.9959)
LISNN [38]	Spike-based BP	99.45	(0.9928, 0.9958)
Spikingjelly [39]	Spike-based BP	99.61	(0.9947, 0.9971)
This work	STDP-based BP	99.67	(0.9954, 0.9976)

**Table 5:** Performance comparison of SNN, SNN with traditional LIN, SNN with adaptive LIN, parallel SNN, parallel SNN with traditional LIN, and parallel SNN with adaptive LIN on the breast ultrasound dataset (*z* = 1.96).

Models	Accuracy (%)	Confidence intervals
Nonparallel SNN without LIN	87.2	(0.8337, 0.9028)
Nonparallel SNN with traditional LIN	89.5	(0.8584, 0.9221)
Nonparallel SNN with adaptive LIN	89.9	(0.8647, 0.9269)
Parallel SNN without LIN	95.3	(0.9257, 0.9703)
Parallel SNN with traditional LIN	98.6	(0.9679, 0.994)
Parallel SNN with adaptive LIN	99.2	(0.9758, 0.9972)

**Table 6:** Performance comparison between the proposed method and the state-of-the-art methods on the breast ultrasound dataset (*z* = 1.96).

Models	Accuracy (%)	Confidence intervals
Google AutoML vision [45]	86.5	(0.8396, 0.8876)
ResNet-18 (28) [46]	85.9	(0.8328, 0.8817)
ResNet-18 (224) [46]	87.8	(0.8534, 0.8993)
ResNet-50 (28) [46]	85.3	(0.826, 0.8757)
ResNet-50 (224) [46]	83.3	(0.8055, 0.8578)
Auto-sklearn [47]	80.8	(0.7786, 0.8338)
AutoKeras [48]	80.1	(0.7719, 0.8278)
DAN [49]	79.2	—
DDC [49]	77.6	—
MADA [49]	79.9	—
DAAN_R18 [49]	80.8	—
DAAN_R50 [49]	81.6	—
MK_DAAN_R50 [49]	83.2	—
This work	99.2	(0.9758, 0.9972)

**Table 7:** Performance comparison of SNN, SNN with traditional LIN, SNN with adaptive LIN, parallel SNN, parallel SNN with traditional LIN, and parallel SNN with adaptive LIN on the Mini-MIAS dataset (*z* = 1.96).

Models	Accuracy (%)	Confidence intervals
Nonparallel SNN without LIN	89.72	(0.8437, 0.9375)
Nonparallel SNN with traditional LIN	91.0	(0.8585, 0.9472)
Nonparallel SNN with adaptive LIN	92.69	(0.8735, 0.9566)
Parallel SNN without LIN	95.23	(0.9044, 0.9744)
Parallel SNN with traditional LIN	95.42	(0.9124, 0.9786)
Parallel SNN with adaptive LIN	98.99	(0.9556, 0.9966)

**Table 8:** Performance comparison between the proposed method and the state-of-the-art methods on the Mini-MIAS dataset (*z* = 1.96).

Models	Algorithms	Accuracy (%)	Confidence intervals
Adaptive thresholding [50]	DuSAT	93.00	(0.8951, 0.952)
Fisher's LDA [51]	FLDA	94.57	(0.9171, 0.9668)
Deep distance metric learning [52]	Stochastic gradient descent	96.7	(0.9398, 0.9808)
Neural network [53]	Self-organizing map	95.20	(0.9208, 0.9692)
Simple logistic with super-resolution reconstruction [54]	Simple logistic	96.70	(0.9398, 0.9808)
GoogLeNet [55]	Error BP	94.67	—
ResNet-101 [55]	Error BP	94.65	—
DenseNet-201 [55]	Error BP	94.47	—
Xception [55]	Error BP	95.17	—
Inception-v3 [55]	Error BP	94.51	—
DisepNet [55]	Error BP	95.60	—
This work	STDP-based BP	98.99	(0.9556, 0.9966)

**Table 9:** Performance comparison of SNN, SNN with traditional LIN, SNN with adaptive LIN, parallel SNN, parallel SNN with traditional LIN, and parallel SNN with adaptive LIN on the BreaKHis dataset (*z* = 1.96).

Models	Accuracy (%)			
	40× (%)	100× (%)	200× (%)	400× (%)
Nonparallel SNN without LIN	86.02	87.3	87.15	88.0
Nonparallel SNN with traditional LIN	87.17	90.25	89.68	88.12
Nonparallel SNN with adaptive LIN	90.45	90.0	92.82	92.55
Parallel SNN without LIN	95.66	94.37	96.06	95.63
Parallel SNN with traditional LIN	96.33	94.47	98.54	96.64
Parallel SNN with adaptive LIN	97.06	97.29	98.60	98.45

**Table 10:** Performance comparison between the proposed method and the state-of-the-art methods on the BreaKHis dataset.

Models	Algorithms	Accuracy (%)			
		40×	100×	200×	400×
AlexNet [56]	Stochastic gradient descent	90.0%	88.4%	84.6%	86.1%
PFTAS + SVM [31]	SVM	81.6%	79.9%	85.1%	82.3%
PFTAS + RF [31]	Random forests	81.8%	81.3%	83.5%	81.0%
PFTAS + QDA [31]	Quadratic linear analysis	83.8%	82.1%	84.2%	82.0%
CSDCNN [57]	Backpropagation	97.1%	95.7%	96.7%	95.7%
Inception-v1 [58]	Asynchronous stochastic gradient descent	89%	92%	94%	90%
MobiHisNet [59]	Backpropagation	91.42%	89.93%	92.70%	85.84%
Backbone [14]	Backpropagation	91.24%	92.96%	95.20%	91.03%
Backbone + Ghost [14]	Backpropagation	96.99%	96.00%	97.58%	96.34%
Backbone + BN [14]	Backpropagation	95.74%	96.16%	97.43%	94.96%
Backbone + MPN-COV [14]	Backpropagation	93.99%	92.48%	96.52%	92.40%
DsHoNet [14]	Backpropagation	97.78%	98.40%	99.01%	97.74%
The ensemble SNN [10]	STDP	98.7%	95.1%	96.7%	97.5%
This work	STDP-based BP	97.06	97.29	98.60	98.45

## Data Availability

The data used to support the findings of this study are included within the article.
